# Age‐specific differences in the magnitude of malaria‐related anemia during low and high malaria seasons in rural Zambian children

**DOI:** 10.1002/jha2.243

**Published:** 2021-06-04

**Authors:** Clement O. Acheampong, Maxwell A. Barffour, Kerry J. Schulze, Justin Chileshe, Ng'andwe Kalungwana, Ward Siamusantu, Keith P. West, Amanda C. Palmer

**Affiliations:** ^1^ College of Health and Human Services, Public Health Program Missouri State University Springfield Missouri USA; ^2^ University of Missouri School of Medicine Patient Centered Care Learning Center Columbia Missouri USA; ^3^ Bloomberg School of Public Health, Department of International Health Johns Hopkins University Baltimore Maryland USA; ^4^ Tropical Disease Research Centre Ndola Zambia; ^5^ National Food and Nutrition Commission Lusaka Zambia

## Abstract

**Background:**

Malaria causes anemia by destruction of red blood cells and inhibition of erythropoiesis.

**Objective:**

We assessed whether the magnitude of the malaria‐specific effect on anemia differs by age, during low and high malaria seasons.

**Method:**

In rural Zambian children participating in a pro‐vitamin A efficacy trial, we estimated differences in the prevalence of anemia (defined as hemoglobin < 110 g/L for children < 60 months. and < 115 g/L in older children) by malaria status and assessed malaria‐age interactions. Regression models (with anemia as the outcome) were used to model malaria‐age interaction in both the low and high malaria seasons, controlling for potential confounders.

**Results:**

Average age was 68 months at baseline (*n* = 820 children). In the low malaria season, anemia prevalence was 29% in malaria‐negative children and 54% in malaria‐positive children (*p* < 0.001), with no malaria‐age interactions (*p* = 0.44). In the high malaria season, anemia prevalence was 41% in malaria‐negative children and 54% in malaria‐positive children (*p* < 0.001), with significant malaria‐age interactions (*p* = 0.02 for anemia). Age‐stratified prevalence of anemia in malaria positive versus negative children was 67.0% versus 37.1% (in children < 60 months); 57.0% versus 37.2% (in 60–69 months.); 46.8% versus 37.2% (in 70–79 months.); 37.0% versus 37.3% (in 80–89 months) and 28.0% versus 37.4% (in 90+ months).

**Conclusions:**

Malarial anemia is most severe in younger children, especially when transmission is intense. Anemia control programs must prioritize this vulnerable group.

## 1 INTRODUCTION

Childhood anemia, defined as low blood hemoglobin, is a global public health problem [1, 2]. Worldwide, anemia affects over 50% of preschool‐aged children, with a disproportionately high prevalence in sub‐Saharan Africa [3]. In severe forms, anemia is a common cause of death [4, 5]. Furthermore, anemia in children has been associated with sub‐optimal cognitive and physical development [6, 7, 8]. The causes of anemia are often multifactorial and are interrelated in a complex way [9]. Historically, global anemia control efforts have predominantly focused on iron deficiency, guided by the premise that iron deficiency (ID) causes almost half of all anemia cases [9]. In recent years, attention to non‐ID related anemia is growing, in part because of accruing evidence suggesting that only ∼30% of anemia is amenable to iron supplementation [10]. Of the other causes of anemia, malaria‐induced anemia is of particular relevance to sub‐Saharan Africa, where malaria is endemic. In this region, malarial anemia is a major cause of sickness and deaths in children [11, 12]. Hence, characterization of the epidemiology of malaria‐related anemia is critical in designing optimal interventions for anemia control.

Malaria‐related anemia is characterized by destruction of parasitized and especially non‐parasitized erythrocytes, and impaired erythrocyte production during malaria episodes [13, 14]. The pathogenesis is complex, and the mechanism of malarial anemia is not fully understood. Because clinical immunity to malaria takes several years of repeated exposure to develop, malaria is typically most severe in young children, whose immune systems are immature [15, 16, 17, 18, 19]. Unfortunately, evidence is lacking on how this age‐specific variation in malaria severity translates into anemia. In this study, we assessed whether the magnitude of the malaria‐specific effect on anemia differs by age, during low and high malaria seasons, among children in rural Zambia, a malaria endemic region. Specific objectives include a) to compare hemoglobin values (and prevalence of anemia) in children by malaria status in both the low and high malaria seasons and b) to determine if age is an effect modifier in the association between malaria and anemia in children.

## 2 METHODS

### 2.1 Study design and participants

This study included 4‐ to 8‐year‐old children from Mkushi District in Central Province, Zambia, an area with a high burden of malaria and anemia [20, 21, 22]. Data and biospecimens were collected as part of a cluster‐ randomized controlled trial, designed to assess the efficacy of pro‐vitamin A carotenoid‐biofortified maize consumption on the vitamin A status of children (registered at clinicaltrials.gov as NCT01695148). Detailed methodology and primary results of the parent trial were published previously [23].

Briefly, we conducted baseline and endline surveys in September 2012 and March 2013, respectively, which corresponded with the low and high malaria transmission seasons, respectively. At both timepoints, we collected socioeconomic data and morbidity history and measured height to the nearest 0.1 cm using a Shorr board, weight to the nearest 0.1 kg with an SECA 874 digital scale and axillary temperature using a digital thermometer. Children presenting with a high fever (axillary temperature > 39.5°C) were referred to the nearest health center. Using a syringe, we collected from each child, ∼7 mL of venous blood into a 10 mL collection tube (Monoject sterile tubes with no additives; Covidien). A malaria rapid diagnostic test (RDT) (05FK50 SD Bioline Malaria Ag P.f; Standard Diagnostics) was used to test for malaria in the field. Children who had positive RDT results during the baseline and endline assessments were treated with artemether and lumefantrine (Coartem; Novartis) in accordance with Zambian guidelines [24]. Thick and thin venous blood films were prepared using 2 and 10 μl of blood, respectively, and venous hemoglobin was assessed using a HemoCue photometer. Blood containing tubes were kept in cooler boxes containing ice packs, allowed to clot, and then transported to field laboratories for separation into serum by centrifugation. Serum was aliquoted into prelabeled cryovials, transported in liquid nitrogen, and stored at ‐80 °C until analyzed. All children were given an insecticide‐treated bed net at baseline.

Ethics approval was obtained from the Institutional Review Board of the Johns Hopkins Bloomberg School of Public Health in Baltimore, Maryland, USA, and the Ethics Review Committee of the Tropical Disease Research Center (TDRC) in Ndola, Zambia.

### 2.2 Laboratory analyses

Thick and thin malaria microscopic slides were read at the TDRC laboratory. Slides were stained with 3% Giemsa, washed, dried, and read by two independent microscopists. Where necessary, a third independent reading was performed to resolve discordant results. Parasite density was estimated using thick films by counting the number of parasites per 200 white blood cells. A slide was considered negative if no parasites were detected after counting 200 oil immersion fields. For each positive thick slide, the corresponding thin film was read to determine the Plasmodium species. Commercial ELISA kits were used to determine serum concentrations of α_1_‐acid glycoprotein (AGP) (Abcam, Cambridge, MA, USA; catalog # ab108854), ferritin (Ramco Laboratories, Inc., Stafford, TX, USA; catalog # S‐22), and soluble transferrin receptor (sTfR; Ramco Laboratories Inc., Stafford, TX, USA; catalog # TFC‐94) at TDRC. We measured C‐reactive protein (CRP) using an Immulite 1000 analyzer (Siemens Medical Solutions Diagnostics, Malvern, PA, USA; catalog # LKCRO1) at JHSPH. We used reverse‐phase high performance liquid chromatography to measure serum retinol concentration at Craft Technologies, Inc., in Wilson, North Carolina, USA. All baseline assessments were repeated at the endline, 6 months later.

### 2.3 Definitions

Anemia was defined as hemoglobin < 110 g/L for children < 60 months and < 115 g/L for children ≥ 60 months [25]. Malaria was defined by either positive RDT, positive microscopy, or both. Malarial anemia was defined as positive test for malaria and concurrent anemia. Acute inflammation was defined as CRP > 5 mg/L, and chronic inflammation was defined as AGP > 1 g/L.

### 2.4 Sample size and power considerations

Because the study intervention had no impact on hemoglobin concentration, we pooled data from all children who had a hemoglobin measurement and malaria microscopy at both time points, regardless of treatment allocation (*n* = 820). With a sample size of 820 (274 anemic cases and 546 non‐anemic cases) and 5% type‐1 error rate, we had 80% power to detect a difference of 10% in anemia prevalence comparing malaria‐positive children and malaria‐negative children.

### 2.5 Statistical analyses

Indicators of nutritional status, socio‐demographic status, inflammations, anemia, and malaria at baseline and endline are summarized as means or proportions (Tables 1 and 2). Paired *t*‐test or McNemar's test was used to compare means and proportions, respectively, between baseline and endline values (Table 2). Linear or logistic regression models with interaction terms were used to evaluate whether the risk of malarial anemia differed by age. We constructed linear regression models with hemoglobin as the outcome and malaria as the primary predictor variable. We constructed logistic regression models with anemia (dichotomized) as the outcome of interest and malaria as the primary predictor variable. For each model type, an age‐malaria interaction term was included to test for potential effect modification by age. In assessing age‐malaria interaction, age was included as a continuous variable. A significant age interaction was defined as a *p* (for interaction) < 0.1. In the event of significant age interactions, plots were constructed to visualize how the association between malaria and anemia (or hemoglobin) changed by age. Ferritin, retinol, and sTfr were log‐normalized.

**TABLE 1:** Socio‐demographic, nutritional status, and morbidity history of Zambian children enrolled during the low malaria season (*n* = 820)

Variable	Number (%)
Household characteristics	
Literate household head	658 (82.1)
Access to electricity	40 (4.9)
Child characteristics	
Age, months^a^	68.3 ± 15.0
Age < 60 months	285 (35.0)
Female	408 (50.9)
Underweight^b^	116 (14.5)
Stunted	230 (28.9)
Morbidity history	
Fever in past 2 weeks	231 (28.6)
Cough in the past two weeks	461 (57.5)
Diarrhea in the past 2 weeks	47 (5.9)

^a^ Arithmetic mean ± standard deviation.

^b^ Underweight and stunting defined as weight‐for‐age Z‐score ← 2 and length‐for‐age Z‐score < ‐2 standard deviation of the WHO Growth Standards (WHO, 2006).

**TABLE 2:** Prevalence of anemia, malaria, and inflammation in Zambian children aged 4–8 years during low‐ and high‐malaria transmission seasons

Indicator	Low malaria season (September 2012, *n* = 820)	High malaria season (March 2013, *N* = 820)	*p*‐value^a^
Hemoglobin, g/L Anemia (%)	117.3 ± 1.3 274 (33.4)	118.4 ± 3.7 330 (40.2)	0.41 <0.001
Malaria^a^ (%)	174 (21.2)	414 (50.5)	<0.001
RDT positive	153 (19.0)	387 (47.1)	<0.001
Microscopy positive	105 (13.0)	173 (21.0)	<0.001
Inflammation			
AGP > 1.0 g/L (%)	363 (44.2)	604 (73.7)	<0.001
CRP > 5.0 mg/L	140 (17.1)	263 (32.1)	<0.001
Ferritin, μg/L^a^	42 ± 3	88 ± 3	<0.001
Low Ferritin (%) ^b^	64 (7.8)	38 (4.6)	0.004
sTfR, mg/L^a^	7.1 ± 1.5	9.2 ± 1.9	<0.001
sTfR > 8.3 mg/L (%)	222 (27.0)	440 (53.6)	<0.001
Retinol, μmol/L	1.01 ± 0.28	1.00 ± 0.32	0.61
Retinol < 0.7 umol/L (%)	88 (10.7)	136 (16.5)	<0.001

Values represent frequency (proportion), means ± SD for hemoglobin, and median (IQR) or AGP and CRP.

Abbreviations: AGP, α_1_‐acid glycoprotein; CRP, c‐reactive protein; RDT, rapid diagnostic test; sTFR, Soluble Transferin Receptor.

^a^ Paired *t*‐test or McNemar's test was used to compare means and proportions, respectively, between baseline and endline values.

^b^ Anemia defined as hemoglobin < 11 g/dl for children < 60 months and < 11.5 g/dl in older children.

^c^ Malaria defined as either RDT positive, microscopy positive, or both.

In the models, malaria was defined on the basis of microscopy alone. There were fewer numbers of children with malaria data at baseline (*n* = 801) compared to endline (820).

For all other tests, statistical significance was set at *p* = 0.05. All data analyses were conducted with STATA 13 software (StataCorp). In adjusted models, iron status (defined by ferritin and sTfR), vitamin A status (defined by serum retinol), and inflammation (defined by CRP and AGP) were included as covariates.

## 3 RESULTS

From 1024 children enrolled in the trial, we included a subset (*n* = 820) who had complete baseline and endline data for hemoglobin, ferritin, sTfR, malaria, CRP, and AGP at both baseline and endline. The children in this subsample were statistically similar to those in the overall trial with respect to age and gender. The mean (± SD) age of the study population was ∼68 months (±15) with ∼35% below the age of 5 years (**Table** **1**). The prevalence of reported fever, cough, and diarrhea at baseline (low malaria season) were 29%, 58%, and 6%, respectively. The prevalence of stunting was 29%. Underweight and stunting were defined as weight‐for‐age Z‐score less than ‐2 and length‐for‐age Z‐score less than ‐2 standard deviation of the WHO Growth Standards respectively (WHO, 2006).

**Table** **2** shows the distribution of anemia, malaria, and inflammation in the low and high malaria transmission seasons. The prevalence of anemia increased from 33% in the low malaria season to 40% during the high malaria season. The prevalence of malaria more than doubled from 21% in the low malaria season to 51% in the high malaria season. Prevalence of acute and chronic inflammation was 17% and 44%, respectively, in the low malaria season, increasing to 32% and 74%, respectively, in the high malaria season.

**FIGURE 1:**
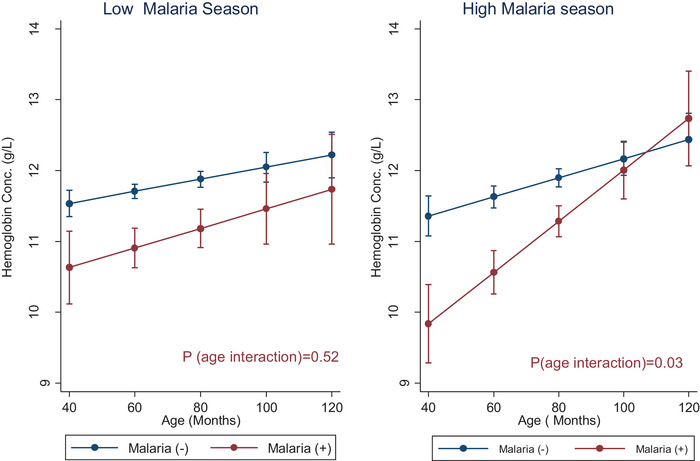
Interaction plots showing age‐differences in hemoglobin concentration during high malaria season in Zambian children, stratified by malaria status

**FIGURE 2:**
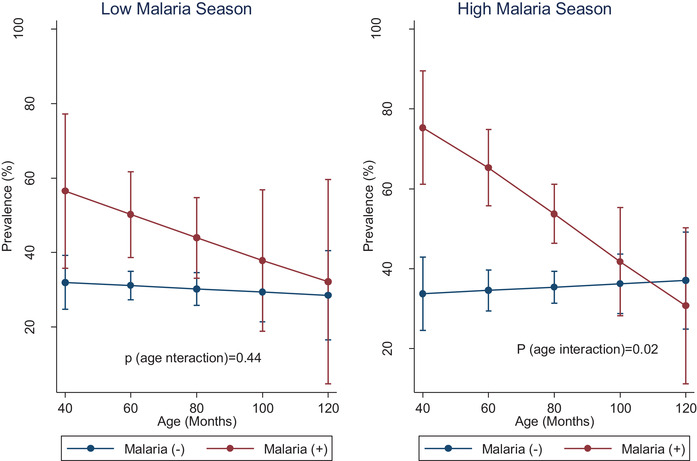
Interaction plots showing age‐differences in anemia during high malaria season in Zambian children, stratified by malaria status

In the low malaria season, there was no statistically significant difference in the hemoglobin concentrations among children with or without malaria after controlling for age, iron status, vitamin A status, and inflammation (**Table** **3** **, *p* <** **0.18)**. In addition, no effect modification by age was observed (*p*‐interaction = 0.45). In the high malaria season, however, both hemoglobin and anemia differed significantly by malaria status, and in addition, a significant age interaction was observed. After adjusting for covariates, mean hemoglobin was 17 g/L lower in children diagnosed with malaria compared to children without malaria (*p* = 0.01). As shown in **Table** **4**, this translated into a nine‐fold increase in the risk of anemia (odds ratio [OR] = 9.32, *p* = 0.02). Furthermore, significant age interactions were observed in the high malaria season (*p* = 0.03 for hemoglobin, and *p* = 0.07 for anemia). Figures 1 and 2 show the age‐interaction plots for hemoglobin and anemia, in both the low and high malaria seasons. In the youngest children (<60 months), anemia prevalence was about 30% higher in those diagnosed with malaria (Figure 2). The magnitude of the difference in anemia prevalence declined gradually until the differences were no longer apparent in the older children. The age distribution of anemia by malaria status is presented in Table S5 (supplemental data). Among children with malaria, anemia prevalence ranged from as high as 67% in children < 5 years to as low as 28% in children > 8 years.

**TABLE 3:** Association between malaria and hemoglobin concentration (g/L) and interactive effects of age during low and high malaria season among Zambian children

	Unadjusted*			Adjusted			
Variables	Mean Hemoglobin	Regression coefficient (95% CI)	*p*‐value for malaria	*p*‐value for malaria‐age interaction	Regression coefficient (95% CI)	*p*‐value for malaria coefficient	*p*‐value for malaria‐age interaction
**Low malaria season (*n* = 801)**							
Malaria negative (*n* = 691)	117.5 ± 1.2	Reference	–	–	Reference	–	–
Malaria positive (*n* = 110)	110.9 ± 1.4	‐10.61 (22.53, 1.31)	0.08	0.53	‐7.47 (‐18.49, 3.54)	0.18	0.45
Covariates							
Ferritin	–	–	–	–	0.6 (‐7.00, 8.0)	0.88	–
Stfr	–	–	–	–	‐12.21 (‐14.20, ‐10.20)	<0.01	–
Age	–	–	–	–	0.91 (0.40, 1.47))	0.01	–
**High malaria season (*n* = 820)**							
Malaria negative (*n* = 629)	118.0 ± 1.6	0.00	–	–	0.00	–	–
Malaria positive (*n* = 197)	111.5 ± 1.8	‐25.27 (‐39.29, ‐11.26)	<0.001	<0.01	‐17.20 (‐30.33, ‐4.08)	0.01	0.03
Covariates							
Ferritin	–	–	–	–	‐40.72 (‐48.33, ‐31.11)	<0.01	–
Stfr	–	–	–	–	‐46.94 (‐63.50, ‐30.38)	<0.01	–
Age	–	–	–	–	1.77 (1.07, 2.49)	<0.01	–

*Differences in mean hemoglobin concentration were tested using linear regression. In unadjusted models, only malaria and age were included as covariates. In adjusted models, concentration of serum ferritin, soluble transferrin receptor, retinol, and inflammation were included as covariates, in addition to malaria and age.

**TABLE 4:** Association between malaria and anemia status and interactive effect of age during low and high malaria season among Zambian children

Variables		Unadjusted*			Adjusted		
	Anemia number (%)	Odds ratio (95% CI)	*p*‐value for malaria	*p*‐value for malaria‐age interaction	Odds ratio (95% CI)	*p*‐value for malaria	*p*‐value for malaria‐age interaction
**Low malaria season (*n* = 801)**							
Malaria negative (*n* = 691)	206 (29.8)	Reference	–	–	Reference	–	–
Malaria positive (*n* = 110)	59 (53.6)	1.66 (‐0.30, 3.62)	0.10	0.52	4.56 (0.60, 34.52)	0.14	0.44
**Covariates**							
Ferritin	–	–	–	–	0.86 (0.74, 1.01)	0.07	–
stfr	–	–	–	–	3.33 (2.23, 5.00)	0.01	–
Age	–	–	–	–	1.00 (0.99, 1.01)	0.707	–
**High malaria season (*n* = 820)**							
Malaria negative (*n* = 629)	219 (35.2)	Reference	–	–	Reference	–	–
Malaria positive (*n* = 197)	111 (56.4)	17.65 (2.98, 104.51)	<0.01	0.03	9.32 (1.48, 58.58)	0.02	0.07
**Covariates**							
Ferritin	–	–	–	–	1.54 (1.36, 1.76)	0.01	–
stfr	–	–	–	–	1.55 (1.21, 1.98)	0.01	–
Age	–	–	–	–	0.99 (0.98, 1.00)	0.26	–

*Differences in anemia status were tested using logistic regression. In unadjusted models, only malaria and age were included as covariates. In adjusted models, concentrations of serum ferritin, transferrin receptor, retinol, and inflammation were included as continuous covariates, in addition to malaria and age.

## 4 DISCUSSION

This study assessed the magnitude of malarial anemia and characterized potential age‐specific trends in the burden of malarial anemia among 4‐ to 8‐year‐old Zambian children, across two malaria seasons. We observed that in both high and low malaria transmission seasons, anemia prevalence was significantly higher among children with malaria. More importantly, we observed that in the high malaria season (but not in the low malaria season), the malaria‐specific effect on anemia was age‐specific. In the youngest age group (<60 months), we observed ∼30 percentage point difference in anemia prevalence between children with and without malaria. However, this difference became narrower with age. Beyond the age of 80 months, this difference was no longer apparent.

The observed high risk of malarial anemia among younger children could be due to a number of factors, including the degree of micronutrient deficiency, outdoor exposure, and malaria‐specific immunity, which improves with age. We previously showed that both iron and vitamin A status influenced malaria and anemia risk in this population [26, 27]. Because nutritional deficiencies are generally higher in younger children, it is plausible that the observed age‐pattern in malarial anemia may in fact be driven by micronutrient deficiencies. However, in this paper, adjustment for both iron and vitamin A status did not change the observed age‐specific patterns in malarial anemia. The more plausible explanation is that the age‐related pattern in malarial anemia is driven by underlying differences in acquired clinical immunity to malaria. In this population, although malaria prevalence increased with age (perhaps because older children engaged in more outdoor activity) [26], the parasite density was generally higher in the youngest children, particularly during the high malaria season (data not shown). This is consistent with previous studies which showed that clinical immunity to malaria develops gradually and after repeated exposures [15]. This reasoning is also supported by the fact that the age‐specific patterns in malarial anemia were observed only in the high malaria season but not the low malaria season. Unfortunately, we did not assess biomarkers of malaria‐specific immunity in this study. Thus, additional evidence is needed to enhance understanding of the observed patterns.

There are several strengths, but also some limitations with the current study. This study is limited by its observational nature, particularly considering the complex etiology of anemia in children. Hemoglobin levels are driven by a complex interplay of nutritional factors (particularly iron metabolism), inflammation and in the context of malaria, by factors which modulate erythrocyte destruction and synthesis [28]. Because these factors are correlated, it is challenging to elucidate cause‐specific effects on anemia from an observations study. To mitigate this potential bias, we adjusted for iron status (using ferritin and sTfR) and inflammation (using CRP and AGP). Another challenge is the lack of data on other factors such as non‐malarial infections, including from helminths [29] and HIV which are both prevalent in this setting [30]. It is worth mentioning that all children participating in this study were provided deworming tablets in accordance with current recommendation. Hence, it is unlikely that our estimates are significantly biased by undiagnosed helminth infections. Furthermore, we did not have data on sickle cell status, a major determinant of anemia in this population [31]. Our study is strengthened by its longitudinal nature, which allowed us to explore how changes in the malaria transmission intensity influenced age‐related pattern in the malarial anemia risk.

In conclusion, our results have important implications for anemia control programs and research in settings with endemic malaria. Our data suggest a need to focus limited resources in preventing and treating malaria in vulnerable young children. In Zambia, preventative strategies such as distribution of insecticide treated bed nets free of charge to children under five are available. In this study, we provided mosquito nets to all children after the baseline survey. Our baseline and endline surveys (data not shown) found that a substantial proportion of children (∼80% in the low malaria season [i.e., before the provision of mosquito nets], and ∼20% in the high malaria season [i.e., after the provision of mosquito nets]) did not sleep under mosquito nets the previous night. Furthermore, because the incidence of malaria was generally high in older children, it plausible that the degree of outdoor exposure is an important risk factor in malaria transmission. Hence, it is important that existing interventions are coupled with educational programs designed to increase awareness about optimal health behavioral practices in the context of malaria prevention and control. Our results suggest that, particularly for young children, anemia control interventions should be integrated into existing malaria control programs. Additional research is needed to understand the pathophysiologic pathways which make young children particularly vulnerable to malarial anemia.

## AUTHORS CONTRIBUTIONS

Clement O. Acheampong drafted the manuscript. Clement O. Acheampong and Maxwell A. Barffour analyzed the data. All authors reviewed and approved the content of the manuscript. Clement O. Acheampong, Maxwell A. Barffour, and Amanda C. Palmer take responsibility for the final content of this paper.

## CONFLICT OF INTEREST

The authors declare that there is no conflict of interest that could be perceived as prejudicing the impartiality of the research reported.

## Supporting information


**Table S5**. Age distribution of anemia prevalence by malaria status in Zambian children during the high malaria season
