# Supplementary material for: Age‐specific differences in the magnitude of malaria‐related anemia during low and high malaria seasons in rural Zambian children
Source: EJHaem. 2021 Jun 4;2(3):349–56. doi: 10.1002/jha2.243 (PMC9175671; doi:10.1002/jha2.243)
Supplement: Supplementary file 1 — Table S5. Age distribution of anemia prevalence by malaria status in Zambian children during the high malaria season [file JHA2-2-349-s001.docx]

**Supplemental Data:**

**Table 5.** Age distribution of anemia prevalence by malaria status in Zambian children during the high malaria season

|  | HEMOGLOBIN (g/L) | | | | ANEMIA (%) | | |
| --- | --- | --- | --- | --- | --- | --- | --- |
| Age Category | N | Malaria Positive | Malaria Negative | P-value (malaria-age interaction) | Malaria Positive (%) | Malaria Negative (%) | P-value (malaria-age interaction) |
| <60 months | 160 | 103  (97, 109) | 113  (110, 116) |  | 66.6  (50.0, 83.1) | 37.1  (27.9, 46.4) |  |
| 60-71 months | 247 | 110  (106, 113) | 115  (114, 117) |  | 57.0  (46.9, 67.1) | 37.2  (32.0, 42.4) |  |
| 72-83 months | 169 | 116  (114, 118) | 118  (117, 119) | 0.03 | 46.8  (39.4, 54.3) | 37.2  (33.3, 41.2) | 0.07 |
| 84-95 months | 167 | 122  (118, 127) | 120  (118, 123) |  | 37.0  (24.0, 49.9) | 37.3  (30.0, 44.6) |  |
| ≥95 months | 77 | 129(121, 136) | 123  (119, 127) |  | 28.0 (9.5, 46.5) | 37.4  (25.6, 49.1) |  |

Values represent mean hemoglobin (95% CI) or prevalence of anemia (95% CI) in different age groups during the high malaria season. Malaria defined as either RDT positive, microscopy positive, or both. Malaria-age interactions were tested in multiple linear regression (for hemoglobin) or multiple logistic regression (for anemia), adjusting for ferritin, soluble transferrin receptor, retinol and inflammation.
